# AMPK-SP1–Guided Dynein Expression Represents a New Energy-Responsive Mechanism and Therapeutic Target for Diabetic Nephropathy

**DOI:** 10.34067/KID.0000000000000392

**Published:** 2024-03-12

**Authors:** Jillian Williquett, Chantal Allamargot, Hua Sun

**Affiliations:** 1 Division of Nephrology, Stead Family Department of Pediatrics, The University of Iowa, Iowa City, Iowa; 2 Carver College of Medicine, The University of Iowa, Iowa City, Iowa; 3 Central Microscopy Research Facility, The University of Iowa, Iowa City, Iowa

**Keywords:** cell and transport physiology, diabetic nephropathy, podocyte, transcription factors

## Abstract

**Key Points:**

- AMP kinase senses diabetic stresses in podocytes, subsequently upregulates specificity protein 1–mediated dynein expression and promotes podocyte injury.
- Pharmaceutical restoration of dynein expression by targeting specificity protein 1 represents an innovative therapeutic strategy for diabetic nephropathy.

**Background:**

Diabetic nephropathy (DN) is a major complication of diabetes. Injury to podocytes, epithelial cells that form the molecular sieve of a kidney, is a preclinical feature of DN. Protein trafficking mediated by dynein, a motor protein complex, is a newly recognized pathophysiology of diabetic podocytopathy and is believed to be derived from the hyperglycemia-induced expression of subunits crucial for the transportation activity of the dynein complex. However, the mechanism underlying this transcriptional signature remains unknown.

**Methods:**

Through promoter analysis, we identified binding sites for transcription factor specificity protein 1 (SP1) as the most shared motif among hyperglycemia-responsive dynein genes. We demonstrated the essential role of AMP-activated protein kinase (AMPK)–regulated SP1 in the transcription of dynein subunits and dynein-mediated trafficking in diabetic podocytopathy using chromatin immunoprecipitation quantitative PCR and live cell imaging. SP1-dependent dynein-driven pathogenesis of diabetic podocytopathy was demonstrated by pharmaceutical intervention with SP1 in a mouse model of streptozotocin-induced diabetes.

**Results:**

Hyperglycemic conditions enhance SP1 binding to dynein promoters, promoted dynein expression, and enhanced dynein-mediated mistrafficking in cultured podocytes. These changes can be rescued by chemical inhibition or genetic silencing of SP1. The direct repression of AMPK, an energy sensor, replicates hyperglycemia-induced dynein expression by activating SP1. Mithramycin inhibition of SP1-directed dynein expression in streptozotocin-induced diabetic mice protected them from developing podocytopathy and prevented DN progression.

**Conclusions:**

Our work implicates AMPK-SP1–regulated dynein expression as an early mechanism that translates energy disturbances in diabetes into podocyte dysfunction. Pharmaceutical restoration of dynein expression by targeting SP1 offers a new therapeutic strategy to prevent DN.

## Visual Abstract

**Figure:**
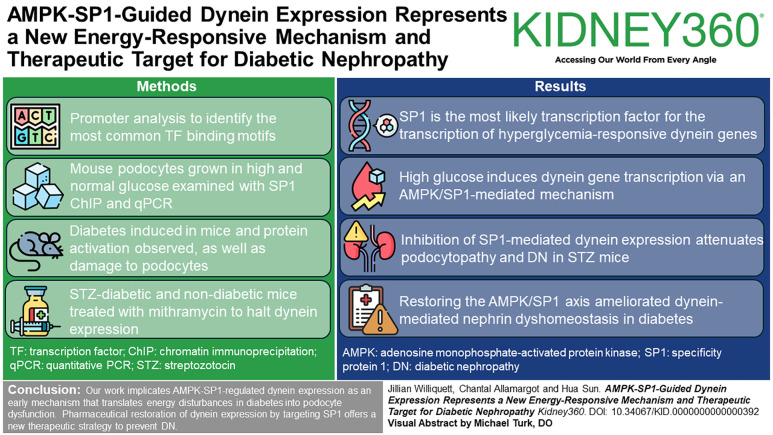

## Introduction

Diabetic nephropathy (DN) is a major complication of diabetes. Thirty to 50% of dialysis patients develop kidney failure secondary to DN, creatine a huge socioeconomic burden.^1–3^ The pathophysiology of DN is considered to be injuries from chronic hyperglycemia and associated glomerular hyperperfusion, which provides the rationale for conventional treatments of DN, including the use of antidiabetic drugs, angiotensin blockers, and sodium–glucose cotransporter-2 inhibitors.^4^ These therapeutic strategies focus on modifying the progression of overt DN in patients who have already developed proteinuria with the underlying pathology of glomerulosclerosis.^1,5^ It is difficult to determine when the earliest kidney injury occurs in patients because most diabetic patients experience a long latency period of 5–8 years after the onset of hyperglycemia and before the onset of microalbuminuria, the earliest clinical presentation of DN.^6–9^ However, during this latency period, irreversible chronic pathology develops.^9^ Therefore, early intervention in diabetic patients at risk of DN holds great promise for improving outcomes.

In diabetes, injuries to podocytes, the epithelial cell component of the glomerular filtration barrier (GFB), develop much earlier than the onset of microalbuminuria.^9^ Our recent work identified a cytoplasmic dynein-mediated trafficking pathway that disrupts proteostasis at the GFB under diabetic conditions.^10^ Dynein is a microtubule-based multiunit motor protein complex that mediates “retrograde trafficking” of membrane proteins from cell surface to cytosol. Dynein-driven mistrafficking of GFB protein is caused by hyperglycemia-induced upregulation of dynein subunits, known as “*hyperglycemia-responsive dynein subunits*.” These include dynein heavy chain (Dync1h1), which functions as an ATPase to generate force for cargoes to slide over microtubules; intermediate chain (Dync1i1), which anchors dynein cargoes; light chain (Dynll1), which assembles the entire dynein complex^11–14^; and dynactin (Dctn1), which activates dynein. In a high-throughput gene transcriptome study of human DN,^15^ synchronized upregulation of these dynein subunits was found to be correlated with albuminuria. Hyperglycemia is the major initiator of DN, and strict glucose control has been shown to delay the progression of DN.^16,17^ Several studies have found that hyperglycemia may cause kidney injury by activating the polyol and hexosamine biosynthesis pathways, activating protein kinase C, or promoting the formation of advanced glycation end products.^18–20^ However, there is no streamlined mechanism that directly connects these complicated hyperglycemia-triggered pathways with kidney injury. In this study, we investigated an energy-responsive transcriptional pathway underlying hyperglycemia-induced transcription of dynein genes.

Bioinformatics analysis of the promoter sequences of the hyperglycemia-responsive dynein genes identified a shared sequence motif characterized by 5′-GGGGCGGGGC-3′ that binds specificity protein 1 (SP1), a two-cysteine, two-histidine zinc-finger family transcription factor (TF), suggesting a unique role for SP1 in mediating synchronous transcription of these dynein subunits. In comparative transcriptome studies, SP1 was identified as a shared TF in both human DN and the eNOS^−/−^ db/db mouse model of DN.^21^ SP1 has been previously reported to play a role in the profibrotic pathway during the progression of mesangial expansion and glomerulosclerosis by promoting the transcription of TGF-*β* and extracellular matrix.^22^ However, these genes were less upregulated than the dynein genes in human DN^15^ and were not significantly correlated with hyperglycemia in rodent models of DN,^21^ suggesting a different mechanism underlying the early pathophysiology of DN.

In this study, we delineated the metabolic pathway responsible for the SP1-guided transcription of dynein and tested whether disrupting this early transcription mechanism can halt the pathogenesis of diabetic podocytopathy and prevent the development of overt DN.

## Methods

### Primers, Antibodies, and Reagents

Primers for chromatin immunoprecipitation (ChIP)-quantitative PCR (qPCR), primers for reverse transcription qPCR, small interfering RNA duplex sequences, antibodies, and chemical compounds are listed in Tables 1–5.

**Table 1:** Primers for chromatin immunoprecipitation-quantitative PCR

Gene	Primers for ChIP-qPCR (Forward Primer/Reverse Primer)
*Dctn1*	TTTTGGAGCAGCGTCTTGGA/GTCACCTAGCAACCACCCAG
*Dynll1*	CTCCGCCTTCAGGGTGTG/GATCTTTCCCCCGCGTTTTG
*Dync1h1*	CTTTTGCGCAGAGTAATGGGT/CTGGAGGCGGGGCTATGT
*Dync1i1*	CTGGGAGTGAAAGTTGCCCAT/GGCAGCGCACACCTTAGTTA
*DCTN1*	TTTTTGGAGCACGCTCTTGG/CATCTA GGGCTTTGCTGG CT
*DYNLL1*	TCGGTAGCGACGGTATCTCT/ GAGTGGGGCGAAATA AGG CA
*DYNC1H1*	TGCCTTTAGGGCGGAGCC/ACCTTCCAGGAGCGATGAGAA
*DYNC1I1*	AGCGTGAGACAGCACATCCT/GACATCCCGCTCCAGTTT ACC

ChIP, chromatin immunoprecipitation; qPCR, quantitative PCR.

**Table 2:** Primers for RT-quantitative PCR

Gene	Primers for qPCR (Forward Primer/Reverse Primer)
*Dctn1*	ATGAGTACGGAGGCAAGCG/AGAATCACGCCCACCCATTTG
*Dynll1*	ATTGCGGCCCATATCAAGAAG/GTGCCACATAACTACCGAAGTTT
*Dync1h1*	AAGCACCTGCGTAAGCTGG/GCGGGTCTGACAGGAACTTG
*Dync1i1*	TAGTCCCAACCCCTATGTCTCC/TGCAGTCGTCTCCTTGTTAATG
*Gapdh*	AGGTCGGTGTGAACGGATTTG/TGTAGACCATGTAGTTGAGGTCA

qPCR, quantitative PCR.

**Table 3:** Small interfering RNA duplex sequences

Target	Cat #	Sense (5′–3′)	Antisense (5′–3′)
Mouse Sp1	sc-29488A	GCAACAUGGGAAUUAUGAAtt	UUCAUAAUUCCCAUGUUGCtt
Mouse Sp1	sc-29488B	CAGUGGCAAUGGUUUCUAAtt	UUAGAAACCAUUGCCACUGtt
Control	sc-37007	UUCUCCGAACGUGUCACGUtt	ACGUGACACGUUCGGAGAAtt

**Table 4:** Antibodies and assay kits

Antibody	Company	Cat #
Mouse antinephrin (G-8)	Santa Cruz	sc-376522
Sheep anti-DCTN1	R&D Systems	AF6657
Rabbit anti-Dync1i1	Aviva	OAAN01119
Rabbit anti-Dync1h1	Bethyl Laboratories	A304-720A-T
Rabbit anti-Dynll1	Thermo Fisher	PA5-97920
Rabbit anti–phospho-AMPK *α*−1 (Thr^172^)	Invitrogen	PA5-35573
Mouse anti-AMPK *α* (*α*1/*α*2)	Santa Cruz	Sc-74461
Rabbit anti–phospho-SP1 (Th^453^)	Bioss Antibodies	BS-12412R
Rabbit anti-SP1	Bethyl Laboratories	A300-134A-T
Mouse anti–*β*-actin-HRP	Santa Cruz	sc-47778
Mouse anti-WT1	Novus Biologicals	NBP2-44607
Rabbit anti-WT1	Invitrogen	MA542786
Rabbit anti-CD34	Invitrogen	PA5-78978
Mouse anti-INF2 antibody	Proteintech	CL488-66910

Assay Kit	Company	Cat #
ChIPAb+ Sp1-ChIP	Millipore-Sigma	17-601
Mouse albumin ELISA kit	Proteintech	KE00076
Creatinine assay kit	Bioassay Systems	DICT015

AMPK, AMP-activated protein kinase; ChIP, chromatin immunoprecipitation; HRP, horseradish peroxidase; INF2, inverted formin 2; SP1, specificity protein 1.

**Table 5:** Chemical compounds

Chemicals	Concentration (*In Vitro*)	Company	Cat #	CAS #
Ciliobrevin D	50 *μ*M	Sigma	250401	1370554-01-0
AICAR	0.5 mM	MedChemExpress	HY-13417A	681006-28-0
CC	50 *μ*M	MedChemExpress	HY-13418A	866405-64-3
MIT	0.1 *μ*M	Santa Cruz	sc-200909	18378-7

Chemicals	Dosing (*in vitro*)	Company	Cat #	CAS #
STZ	150 mg/kg i.p.×one dose	MedChemExpress	HY-13753	18883-66-4
MIT	0.25 mg/kg twice weekly×eight doses	Santa Cruz	sc-200909	18378-7

AICAR, 5-aminoimidazole-4-carboxamide ribonucleoside; CAS, chemical abstracts service; CC, compound C; i.p., intraperitoneal; MIT, mithramycin; STZ, streptozotocin.

### Cell Culture and Experiments

The mouse podocyte cell line was maintained in Roswell Park Memorial Institute 1640 medium with 10% FBS, 1% insulin–transferrin–selenium supplement (Gibco, Grand Island, NY), and 50 IU/ml penicillin–streptomycin solution. Primary human podocytes were purchased from Celprogen Biotechnology Company (Celprogen 36036-08), cultured in precoated flasks with Human Podocyte Cell Culture Extracellular Matrix (Celprogen E36036-08) and maintained in complete medium with serum (M36036). Cells were cultured in media containing normal glucose (glucose 5.5 mM with mannitol added to maintain the same osmolality) or high glucose (HG, glucose 30 mM alone), with chemical or genetic interventions on AMP-activated protein kinase (AMPK) or SP1.

### Live Cell Imaging and Kymograph Analysis

As described in our previous study,^10,14^ nephrin endocytosis was induced by incubating podocytes with antinephrin antibody (Santa Cruz sc-376522), followed by an Alexa Fluor 594-conjugated anti-mouse secondary. Time lapse imaging was captured under a Leica SP8 Confocal microscope every 5 seconds for 10 minutes in three independent experiments. Kymograph was generated using the KymographClear plugin of Fiji software. The fraction and the average velocity of the retrograde, anterograde, and static trafficking events were quantified using KymographDirect plugin.

### ChIP-qPCR

**Figure 1:**
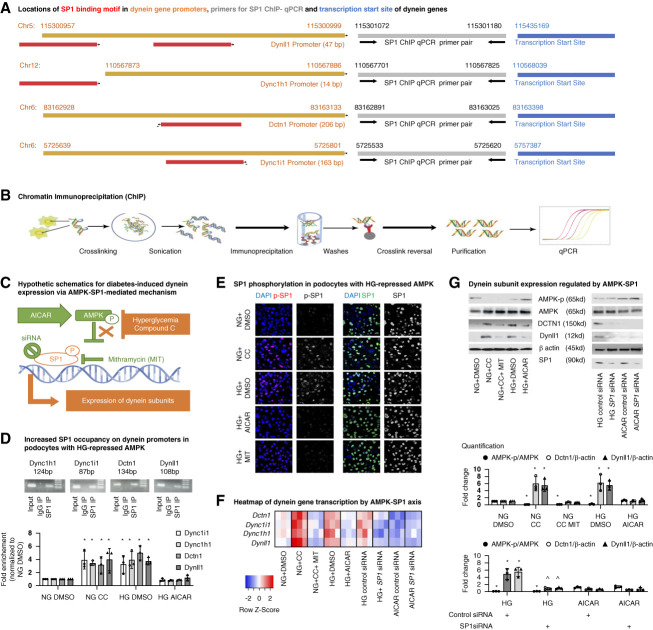
**Hyperglycemia induces dynein gene expression *via* an AMPK/SP1-dependent mechanism.** (A) Hyperglycemia-responsive dynein genes share binding motif for SP1 TF. SP1 binding motifs (red) identified in genes encoding mouse dynein subunits, using SwissRegulon tools. The promoter sequences for each individual dynein gene and their GenBank locations are highlighted in orange. ChIP primers (gray) were designed close to the SP1 binding motif and the translation starting sites (blue). (B) Workflow of crosslinking SP1 ChIP. (C) Hypothesis and research design of hyperglycemia-induced dynein gene expression *via* an AMPK/SP1 axis. Hyperglycemia suppresses AMPK and disinhibits SP1, which subsequently initiates the transcription of dynein subunits. (D) The fold enrichment (=2^(Ct IgG−Ct SP1)^) of dynein gene locus sequences immunoprecipitated with SP1 was quantified by ChIP-qPCR. The values were normalized to that of NG. The PCR products were confirmed by running an agarose gel. (E) SP1 activity reflected by the nuclear location of phosphorylated SP1 in podocytes with different treatments, related to the unchanged total SP1 (NG; HG; CC, an AMPK inhibitor; AICAR: an AMPK agonist). Medium containing 0.3% DMSO served as a negative control for chemical intervention. (F) Relative quantification of dynein gene transcription using Gapdh as a housekeeping gene was normalized to that of NG and was compared in a heatmap. (G) Protein levels of representative dynein subunits in podocytes with different treatments were examined using Western blot. The corresponding AMPK activity was expressed as the Thr^172^ phosphorylated to total AMPK ratio (AMPK-p/AMPK). The log OD values against the *β*-actin housekeeping protein were normalized to that of NG. *n*=3, *P < 0.05 versus NG; ^*P* < 0.05 SP1 siRNA versus control siRNA. AICAR, 5-aminoimidazole-4-carboxamide ribonucleoside; AMPK, AMP-activated protein kinase; CC, compound C; ChIP, chromatin immunoprecipitation; HG, high glucose; NG, normal glucose; OD, optical density; qPCR, quantitative PCR; siRNA, small interfering RNA; SP1, specificity protein 1; TF, transcription factor.

According to the manufacturer's instructions for the SP1 ChIP kit (Figure 1B), chromatin samples were prepared from sonicated podocyte lysates following formalin-induced crosslinking, and normal rabbit IgG and anti-SP1 were used with A/G protein beads preabsorbed with herring sperm DNA to precipitate DNA/protein complexes. Soluble chromatin was used as an input. SYBR qPCR was conducted to target the promoter regions of dynein genes, and the results are represented as Fold Enrichment=2^(Ct IgG−Ct SP1)^.^23^

### Animal Experiment

Eight-week-old C57BL/6J wild-type mice (male) received a single intraperitoneal (i.p.) injection of streptozotocin (STZ) dissolved in 0.05 M sodium citrate buffer (pH=4.5) at a dose of 150 mg/kg body weight.^24,25^ Age-matched vehicle control mice received i.p. injections of sodium citrate buffer of the same volume and regimen. Mithramycin (MIT) was administered to both nondiabetic mice and diabetic mice (2 weeks after the STZ injection) at the dosing regimen of 0.25 mg/kg twice weekly×eight doses *via* i.p. injection.^26^

### Immunofluorescent Staining and Quantification

In paraffin-embedded mouse kidney sections, dynein subunits, phosphorylated SP1, and nephrin were coimmunofluorescent stained with podocyte markers (WT1, inverted formin 2). Using the Fiji ImageJ analysis tool, the media fluorescent intensity for podocyte-specific levels of nephrin, dynein subunit, and phosphorylated SP1 were quantified for comparison among mice with different treatments. Using the Colo2 plugin of the Fiji ImageJ software, nephrin-Dynll1 colocalization was quantified as the Manders overlap coefficient, as described in our previous work.^10^

### Scanning Electron Microscopy

Each kidney cortex was cut into small cubes, immersed, and fixed in Karnovsky's fixative composed of 1.5% glutaraldehyde and 1.6% paraformaldehyde buffered with 0.1 M sodium cacodylate buffer, pH 7.2. It was then treated with a 1% osmium tetroxide in 0.1 M sodium cacodylate buffer, dehydrated, and dried using a CO_2_ critical point dryer. The specimens were then mounted on aluminum scanning electron microscopy (SEM) stubs with carbon tape, sputter-coated with gold and palladium in an Emitech Sputter Coater K550, and imaged using a Hitachi S-4800 SEM.

### Statistical Analyses

Data analyses were performed using GraphPad Prism 9. Immunofluorescence signal analysis and densitometric analysis of the immunoblots were performed using the Fiji/ImageJ software. Data are expressed as mean±SEM. One-way ANOVA was used for comparisons among multiple groups, and a *post hoc q* test was used to compare the differences between groups. In a two-tailed test, *P*<0.05 is considered significant.

### Study Approval

The animal protocol was approved by the Institutional Animal Care and Use Committee of the University of Iowa and was in accordance with the National Institutes of Health guidelines for the use of live animals.

## Results

### SP1 is the Most Likely TF for the Transcription of Hyperglycemia-Responsive Dynein Genes

Using *SwissRegulon* tools for regulatory genomics and predicted TF-binding sites,^27^ we found that SP1 is the TF with the most common binding motif in the promoters of the hyperglycemia-responsive mouse dynein genes *(Dync1h1*, *Dync1i1*, *Dynll1*, and *Dctn1*, Figure 1A) and their human orthologs (Supplemental Figure 1A), suggesting that SP1 is a key TF that mediates synchronous transcription of dynein genes in diabetes.

### HG Induces Dynein Gene Transcription *via* an AMPK/SP1-Mediated Mechanism

Using SP1 ChIP followed by qPCR, we demonstrated an enrichment of promoter sequences for *Dync1h1*, *Dync1i1*, *Dynll1*, and *Dctn1* immunoprecipitated with SP1 in mouse podocytes grown in HG, compared with cells cultured under normal glucose conditions (Figure 1D). This change corresponded to increased mRNA transcription and protein expression of dynein subunits (Figure 1, F and G). These changes can be reversed by chemical or genetic inhibition of SP1 using MIT, a selective chemical inhibitor that competitively displaces SP1 from its binding sequences. HG-induced transcriptional activation of SP1 was also reflected by the increased nuclear levels of Thr^453^-phosphorylated SP1,^28^ relative to the stable level of total SP1 (Figure 1E).

We examined the role of AMPK, a direct cellular energy sensor, in SP1-guided dynein expression induced by HG. First, we found that HG-induced SP1 activation and SP1-directed dynein expression could be replicated in cells treated with compound C, a selective inhibitor of AMPK that mimics repressed AMPK in hyperglycemia. By contrast, HG-upregulated dynein genes can be restored by 5-aminoimidazole-4-carboxamide ribonucleoside (AICAR), an activator of AMPK. Altered AMPK activity in cells was confirmed by measuring the Thr^172^ phosphorylated to total AMPK-ratio ^29^ (AMPK-p/AMPK, Figure 1G). Second, SP1-directed dynein expression following the direct repression of AMPK by compound C could be rescued by MIT. SP1 knockdown rescued HG-induced dynein overexpression, but it had no effects on dynein expression when AMPK is activated by AICAR (Figure 1, F and G), suggesting SP1-mediated transcription of hyperglycemia-responsive dynein subunits is a consequence of AMPK repression.

The AMPK-SP1–regulated transcription of hyperglycemia-responsive dynein subunits was validated in human podocytes (Supplemental Figure 1, B and C).

### Inhibition of SP1-Mediated Dynein Expression Attenuates Podocytopathy and DN in STZ Mice

**Figure 2:**
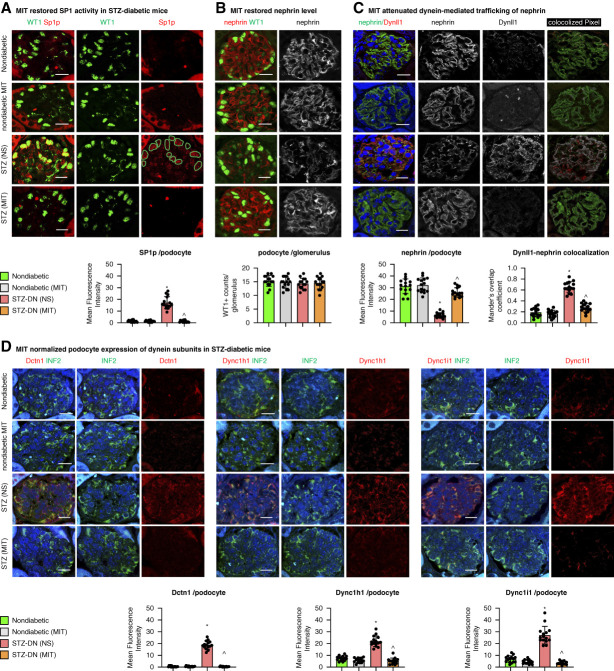
**MIT restored SP1-mediated dynein expression and dynein-mediated nephrin homeostasis in STZ-induced diabetic mice.** Type 1 diabetes was induced in 8-week-old male C57BL/6J mice by single high dose injection of STZ (150 mg/kg, i.p.). Two weeks after the STZ injection, MIT diluted in NS was given *via* i.p. injection at the dose of 0.25 mg/kg, twice weekly for a total of eight doses. Vehicle controls received NS injections. Nondiabetic mice that received MIT injections were included in this study to exclude nephrotoxicity of MIT at this regimen. In STZ-induced diabetic mice, MIT restored SP1 activity (reflected by immunostaining of Thr^453^ phosphorylated SP1 costained with podocyte nuclear marker WT1 (A) reduced nephrin protein (B) and dynein-mediated mistrafficking of nephrin (reflected by increased Dynll1 colocalizing with nephrin, C), and the upregulated expression of dynein subunits (costained with podocyte cytosol marker INF2, D). Mean fluorescence intensity of dynein subunits per podocyte was quantified for comparison. *n*=15 (three glomeruli or three interstitial areas per section×five mice). *P < 0.05 versus nondiabetic mice, ^*P* < 0.05 versus STZ-induced diabetic mice treated with NS. Scale bar: 20 *μ*m. INF2, inverted formin 2; i.p., intraperitoneal; MIT, mithramycin; NS, normal saline; STZ, streptozotocin.

**Figure 3:**
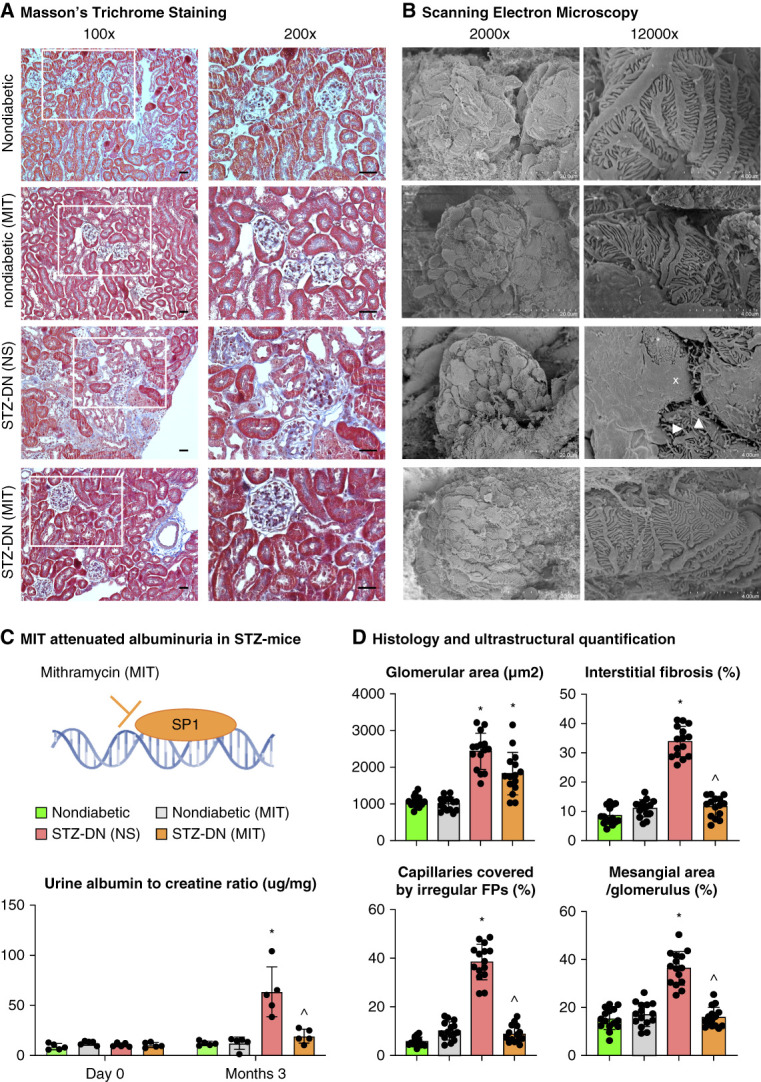
**MIT attenuated podocytopathy and slowed the development of overt nephrosis in STZ-induced diabetic mice.** Masson trichrome staining (A, scale bar 50 *μ*m) and SEM of mouse kidney tissue (B, arrowhead: microvillus transformation, asterisk: podocyte detachment, X: foot process effacement). (C) MIT reduced the urine albumin-to-creatinine ratio in STZ-induced diabetic mice but did not cause albuminuria in nondiabetic mice (*n*=5). (D) The histologic and ultrastructural features of the kidney are quantified as glomerular area, percentages of interstitial fibrosis, percentages of mesangial expansion per glomerulus, and percentages of glomerular capillaries covered by irregular foot processes. *n*=15 (three glomeruli or three interstitial areas per section×five mice), *P < 0.05 versus nondiabetic mice, ^*P* < 0.05 versus STZ-induced diabetic mice treated with NS. SEM, scanning electron microscopy.

In a mouse model of STZ-induced type 1 diabetes, we demonstrated SP1 activation in glomerular podocytes by showing increased immunostaining of Thr^453^-phosphorylated SP1 in podocyte nucleus, which was reversed by treating the animals with i.p. injections of MIT (Figure 2A). This corroborates the synchronized upregulation of hyperglycemia-responsive dynein subunits that are colocalizes with inverted formin 2, a dynein regulating protein that is highly selectively expressed in podocyte cytosol (Figure 2D). Diabetic mice developed albuminuria at 3 months (Figure 3C), resulting from podocyte injury characterized by foot process effacement, microvillous transformation, and detachment, as shown under SEM (Figure 3B). Both the disturbed ultrastructure of podocytes and albuminuria were attenuated by administering MIT in STZ mice compared with the treatment control. Quantification of histologic features in the kidneys of STZ mice showed that MIT treatment prevented the development of mesangial expansion and interstitial fibrosis, pathologic features of overt DN (Figure 3D). Nondiabetic mice received MIT injections did not develop albuminuria or obvious kidney pathology.

### Restoring the AMPK/SP1 Axis Ameliorated Dynein-Mediated Nephrin Dyshomeostasis in Diabetes

**Figure 4:**
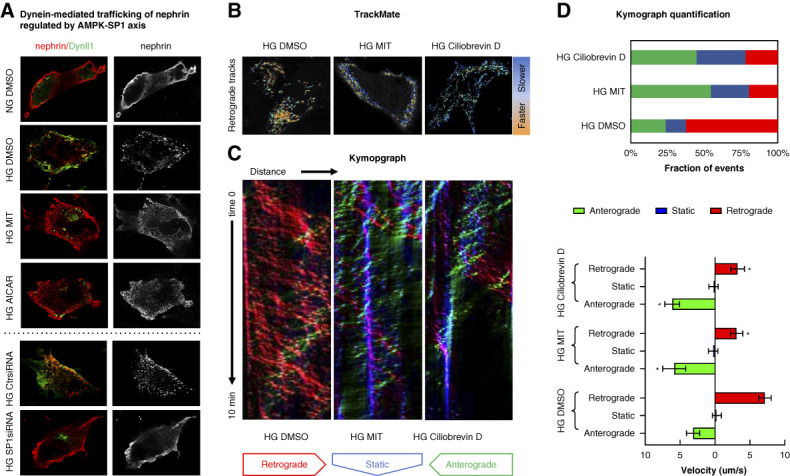
**HG impaired nephrin proteosis *via* AMPK/SP1-regulated dynein expression.** (A) Coimmunostaining of nephrin and Dynll1 in podocytes cultured under different conditions: NG (NG+0.3% DMSO); HG (HG+0.3% DMSO); HG in the presence of MIT (0.1 *μ*M MIT); or AICAR (0.5 mM). (B–D) Nephrin expressed on podocyte surface underwent crosslink-induced endocytosis induced by fluorophore-labeled antinephrin. The postendocytic trafficking of nephrin in HG-cultured podocytes in the presence of MIT or Ciliobrevin D (50 *µ*M) versus control cells (HG+DMSO) was visualized using live cell imaging and analyzed using the TrackMate and KymographClear plugins of Fiji/ImageJ software. With TrackMate (B), faster retrograde trafficking of nephrin (from surface membrane to cytosol) is displayed in warmer colors, and slower tracks are displayed in cooler colors. In the Kymograph (C), the retrograde, static, and anterograde (from cytosol to surface membrane) tracking events are displayed in red, blue, and green. (D) The fraction and velocities of the trafficking events were quantified using KymographDirect software. *P < 0.05 versus HG+DMSO.

Nephrin forms the slit diaphragm, the molecular sieve of the kidney, *via* its extracellular domain. Therefore, accurate targeting of nephrin protein to the surface of podocytes is an important functional marker of GFB. Our previous study showed enhanced dynein-mediated mistrafficking in diabetes caused podocytopathy by diverting nephrin from surface trafficking to proteolysis pathways.^10^ We found that HG treatment increased dynein expression, which subsequently impaired the surface distribution of nephrin in podocytes. These changes can be restored by AICAR, MIT, or small interfering RNA-mediated knockdown of SP1 (Figure 4A). Using live cell imaging, we observed faster and prominent retrograde trafficking of nephrin (from surface to cytosol) in podocytes growing in HG (Figure 4B, tracks in warm colors exhibited by *TrackMate*). By quantifying the trafficking trajectories in Kymograph (Figure 4C), we demonstrated increased fraction and velocity of the retrograde trafficking events in HG, which could be reversed by MIT. The effect of MIT is similar to that of Ciliobrevin D, a direct dynein inhibitor (Figure 4D).

*In vivo*, compared with the linear distribution of nephrin in nondiabetic mouse podocytes, we observed a depleted nephrin with segmented distribution in diabetic mouse kidneys (Figure 2B and Supplemental Figure 2), but it is not due to podocytes loss at this early stage of DN. The nephrin dyshomeostasis in diabetic mice corresponded to the enhanced dynein-mediated trafficking of nephrin reflected by increased Dynll1 colocalizing with nephrin (Figure 2C).^10,14^ These changes were restored by MIT-normalized dynein expression.

## Discussion

Our recent transcriptome analysis identified upregulated dynein genes in the early stages of DN, which correlates with the severity of hyperglycemia and kidney injury. The dynein genes encode subunits that are key to the integrity, motor force, cargo loading, and activity of the dynein transport complex, implicating a role for dynein in the pathogenesis of DN. Further mechanistic studies underscored a new dynein-driven mechanism of diabetic podocytopathy, in which synchronous upregulation of dynein subunits in response to hyperglycemia results in enhanced dynein-mediated mistrafficking and dyshomeostasis of nephrin, leading to podocyte injury.^10^ Investigating how diabetic stresses drive the upregulation of dynein will help us dissect potential therapeutic targets to remodel the pathogenesis of DN.

To understand the transcriptional signature of hyperglycemia-responsive dynein genes underlying early podocyte injury, we identified SP1 as the most likely TF because it binds to the most common motifs in the promoters of all these dynein genes. Using the ChIP assay, we demonstrated increased SP1 enrichment in dynein gene promoters in HG-treated podocytes, leading to their transcription and protein expression. In both cultured podocytes and the STZ-induced type 1 diabetes mouse model with hyperglycemia as the major pathologic factor, we demonstrated activation of SP1 transcription activity and subsequent increased expression of the hyperglycemia-responsive dynein subunits. Furthermore, we demonstrated the essential role of SP1 in HG-induced dynein gene expression and the resultant dynein-mediated mistrafficking of nephrin because these changes can be reversed by specific gene silencing of SP1 or by MIT, a chemical inhibitor of SP1.

The activation of SP1-mediated transcription requires post-translational modifications, such as phosphorylation or glycosylation.^28,30–33^ We showed that HG increased Thr^453^ phosphorylation of SP1, which activates its transcriptional activity. However, the mechanism by which hyperglycemia induces the SP1-mediated transcription of dynein genes is unknown. Studies in endothelial and mesangial cells reported that the phosphorylation activation of SP1 can be induced by various diabetes stresses, such as hyperglycemia, mechanical shear,^30,34^ vascular endothelial growth factor,^32^ and angiotensin II,^35^ and it is mediated *via* complex cascades of kinases involved in diabetes pathways.^31,36–38^ Accumulated evidence shows that AMPK, a direct cellular energy sensor, potentially connects diabetes-responsive kinases/pathways^39–41^ with SP1.^42,43^ Therefore, we hypothesized that *AMPK-regulated SP1 conveys the diabetic metabolic signaling that influences podocyte pathophysiology*. By chemically manipulating AMPK activity in cultured podocytes, we demonstrated that direct repression of AMPK replicates the effects of HG in inducing SP1 activation and SP1-dependent expression of dynein subunits. By contrast, these HG-induced changes were reversed by reactivating AMPK using AICAR. Moreover, AICAR-induced activation of AMPK rescued surface trafficking of nephrin, which was impaired by HG-upregulated dynein.

The diabetes-responsive SP1-targeting genes in different kidney cells remain to be elucidated. Previous studies in mesangial cells have emphasized the role of SP1 in the transcription of profibrotic genes for mesangial expansion and extracellular matrix production, characterizing the late-stage pathology of DN.^22,44^ Our study, on the other hand, identified a new set of dynein-encoding genes as key SP1-targeting genes responsible for early-stage diabetic podocyte injury and delineated how hyperglycemia induces podocytopathy *via* the AMPK-SP1-dynein pathway. This pathway directly senses the energy changes caused by hyperglycemia and transduces metabolic signals to enhance the expression of dynein, which promotes dynein-mediated podocyte injury. While inhibitors of dynein subunits are still under pharmacokinetic and pharmacodynamic investigation for *in vivo* use, our findings offer alternative molecular targets for the intervention of dynein-driven pathogenesis of diabetic podocyte injury, especially AMPK and SP1. Agonists of AMPK (AICAR and metformin) have been shown to provide protective effects in alleviating podocyte injury in mouse models of type 1 diabetes.^42,45^ Although there has been no SP1-targeting therapy for DN, its therapeutic potential is supported by genetic epidemiology data implicating its role in the pathogenesis of both type 1 and type 2 diabetic kidney injury. In a meta-analysis of DN gene expression datasets, SP1 was identified as one of the top TFs with differentially expressed genes enriched in DN.^46^ A single nucleotide polymorphism was found to alter the sequence of the SP1 binding site in the promoter region of target genes associated with DN among type 1 diabetic patients in the Genetics of Kidneys in Diabetes Study,^47^ as well as in type 2 diabetic patients of Tunisian Arab origin.^48^ Intriguingly, we found that HG-disturbed trafficking of nephrin can be rescued by MIT-inhibited SP1 transcription of dynein, equivalent to the effect of Ciliobrevin D, an antagonist for dynein. MIT and its analogs are pharmaceutically available and have been used to treat human malignancy and non-neoplastic animal models of ischemic neuropathy^49^ and myocardiopathy,^50^ providing a rationale for using MIT to interfere with SP1-promoted dynein expression and activation.

We tested whether inhibiting SP1-directed transcription of dynein can rescue DN *in vivo* and showed that MIT treatment of STZ-induced diabetic mice indeed rescued the damaged morphology and filtering function of podocytes by preventing upregulated dynein expression, accompanied by an alleviation of albuminuria and late DN pathology, such as mesangial expansion, glomerular fibrosis, and interstitial fibrosis. In addition, the same dosing regimen of MIT did not cause obvious albuminuria or nephrotoxicity in the nondiabetic mice. MIT is an antineoplastic drug used to treat human cancers or hypercalcemia of metastatic bone disease/Paget bone disease.^51,52^ When used with these indications requiring high or repeated doses, MIT-related nephrotoxicity was reported, especially in patients with baseline kidney disease or predisposed to renal tubular injury.^51^ Pharmacokinetics study found rapid uptake of MIT by tubular cells,^53^ making them more susceptible to toxicity compared with other types of kidney cells. The human equivalent dose of MIT required to counteract dynein-driven diabetic podocytopathy in our mouse model is only 20% of the doses required to treat human cancer. Therefore, fine-tuning the doses and choosing MIT analogs with less toxicity^54^ to control the expression of specific genes in certain types of cells may offer a better therapeutic window for SP1-targeting therapy. SP1, a TF with broad targets, likely regulates transcription in gene-specific and cell type-specific manners. While a smaller dose of MIT suppresses the “pathogenic” upregulation of dynein expression and protects podocytes, a higher or toxic dose may impair the “essential” expression of certain SP1-targeting genes to maintain the function of tubular cells (for instance, hypoxia inducible factor 1 which regulates the hypoxic adaptation^55^ or sphingosine kinase 1 which maintains survival against ischemia/reperfusion injury^56^). Conversely, the broad targets of SP1 extend the potential of MIT analogs from antineoplastic to anti-inflammatory, antireactive oxygen species and antifibrotic effects in developing new treatments for neurodegenerative disease, ischemic cerebrocardiovascular disease,^49,50,57,58^ as well as epithelial–mesenchymal transition after ischemia–reperfusion injury of the kidney.^59^ Therefore, while exploring the protective efficacy of targeting SP-regulated gene transcription in glomerular disease, it is equally important to understand how SP1 regulates gene expression in renal tubular cells, to optimize the therapeutic window of SP1-targeting strategies.

In summary, our work delineates how the AMPK-regulated SP1 pathway leads to diabetes-responsive transcription of dynein genes, provides a better understanding of the initial steps of dynein-driven pathogenesis of DN, and identifies a new molecular target for remodeling dynein-mediated pathology *in vivo*. In addition to DN, SP1-mediated gene transcription and dynein-mediated pathology have been reported in diabetic complications in various organ systems,^60,61^ such as retinopathy^62,63^ and neuropathy.^64,65^ The cause–effect relationship between SP1 and dynein in these organs has not been established in these tissues. By expanding our models from podocytes to other target cells of diabetes, we may provide insights into a potentially common energy-responsive AMPK-SP1-dynein–driven pathogenesis of multiple diabetic complications and the value of pharmaceutically remodeling this pathway to manage them.

## Supplementary Material

## Data Availability

All data is included in the manuscript and/or supporting information.

## Supplemental Material

Supplemental Figure 1. Hyperglycemia induces dynein gene expression *via* an AMPK/SP1-dependent mechanism in human podocytes.

Supplemental Figure 2. Coimmunostaining of nephrin with an endothelial marker in diabetic mouse kidney sections.
