# Supplementary material for: AMPK-SP1–Guided Dynein Expression Represents a New Energy-Responsive Mechanism and Therapeutic Target for Diabetic Nephropathy
Source: Kidney360. 2024 Mar 12;5(4):538–49. doi: 10.34067/KID.0000000000000392 (PMC11093544; doi:10.34067/KID.0000000000000392)
Supplement: Supplementary file 1 [file kidney360-5-538-s001.pdf]

Supplemental Material

Table of contents

Supplementary Figure 1

Supplementary Figure 2

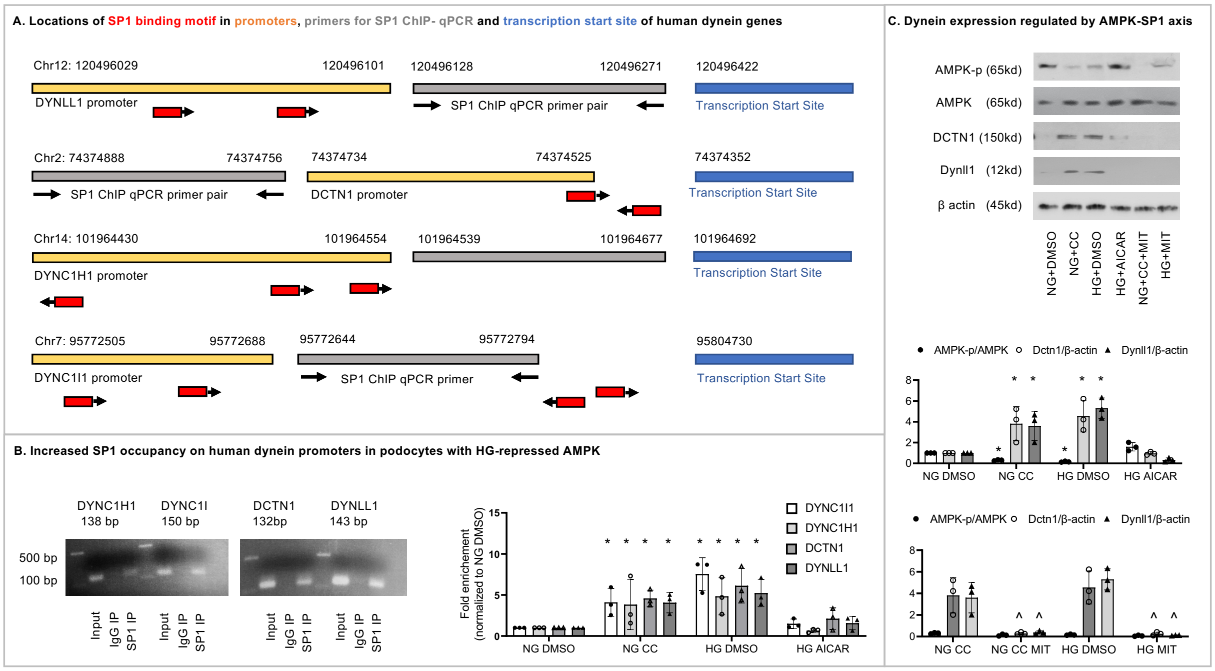

Supplementary Figure 1. Hyperglycemia induces dynein gene expression via an AMPK/SP1-dependent mechanism in human podocytes.

A. Hyperglycemia-responsive human dynein genes share binding motifs for SP1 transcription factor. The SP1 binding motifs (red) were identified in genes encoding human dynein subunits, using *SwissRegulon* tools. The promoter sequences for each individual dynein gene and their GenBank locations are highlighted in orange. ChIP primers (gray) were designed close to the SP1 binding motif and the translation starting sites (blue). **B.** Celprogen human podocytes cultured under different conditions were processed for SP1 ChIP-qPCR. NG: normal glucose; HG: high glucose; CC: compound C, an AMPK inhibitor; AICAR: an AMPK agonist; medium containing 0.3% DMSO served as a negative control for chemical intervention. Relative quantification of fold enrichment ($=2^{(\text{Ct IgG-Ct SP1})}$) of dynein gene locus sequences immunoprecipitated with SP1, normalized to that of NG+0.3%DMSO for comparison. The appropriate sizes of the PCR products were confirmed by running an agarose gel. **C.** Expression of representative dynein subunits in human podocytes with different treatments were examined by western blot. The log optical density (OD) values against the β-actin housekeeping protein, and the AMPK activity was expressed as Thr^{172} phosphorylated AMPK-to-total AMPK ratio (AMPK-p/AMPK). The quantitative values were normalized to that of NG (+DMSO). n = 3, *$p < 0.05$ vs. NG+DMSO; ^$p < 0.05$ cells treated with MIT vs. without MIT.

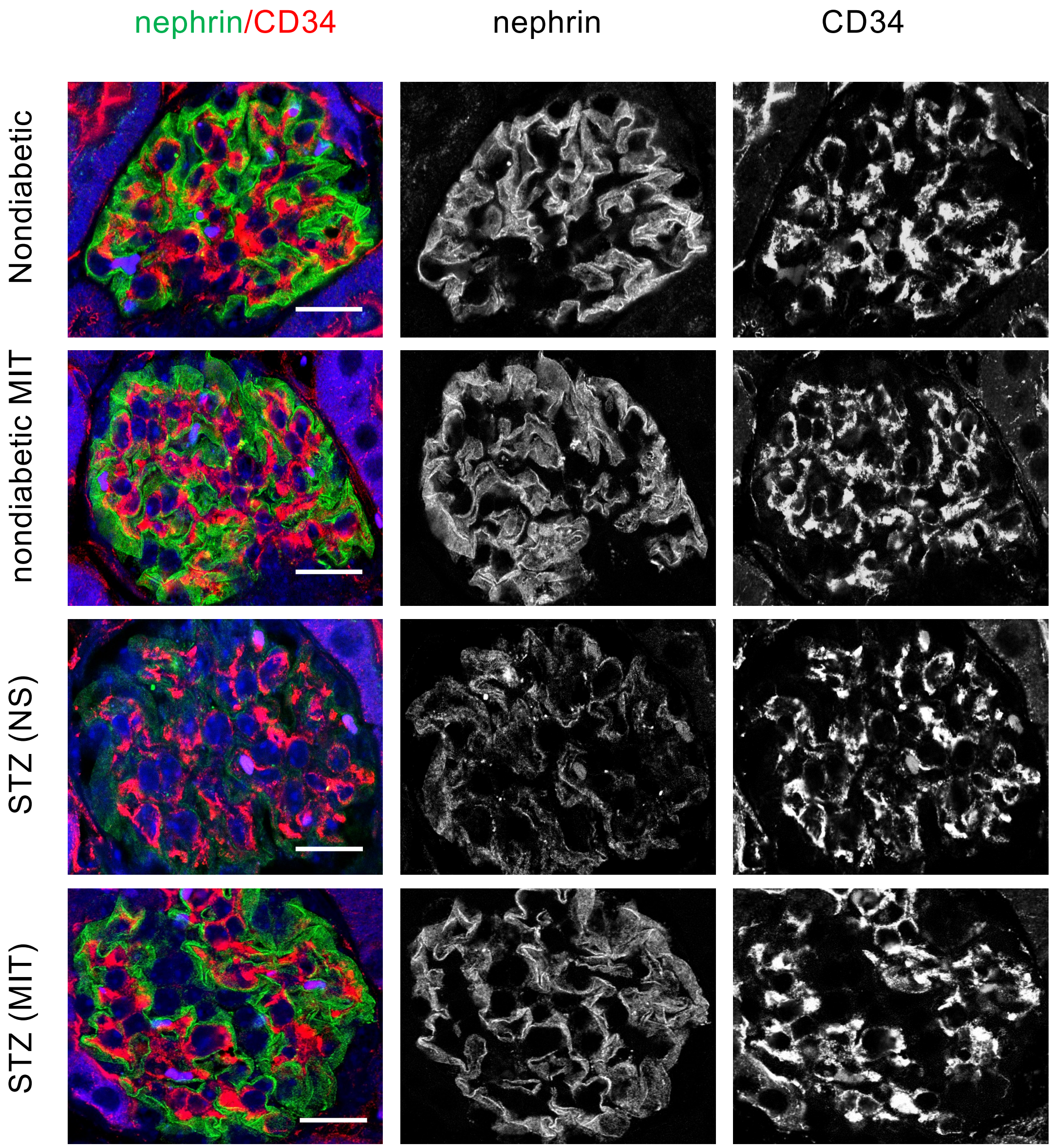

Supplementary Figure 2. Co-immunostaining of nephrin with an endothelial marker in diabetic mouse kidney sections.

Co-immunofluorescent staining of nephrin (mouse anti-nephrin followed by Alexa Fluro 647-conjugated anti-mouse IgG, red channel) with an endothelial marker CD34 (rabbit anti-CD34 followed by Alexa Fluro 488 conjugated-anti-rabbit IgG, green channel) in kidney sections of nondiabetic mice, nondiabetic mice treated with mithramycin (MIT), STZ diabetic mice treated with NS or MIT. Scale bar: 20 μm.
